# One-Year Results of Orthokeratology and Defocus Incorporated Multiple Segment Lenses in French Children for Myopia Control

**DOI:** 10.7759/cureus.83106

**Published:** 2025-04-28

**Authors:** Florent Boulanger, Julien Poret, Pan Liu, Benjamin Jany, Emmanuel Bui Quoc, Thi Ha Chau Tran

**Affiliations:** 1 Ophthalmology, Amiens University Hospital, Amiens, FRA; 2 Medical Image Processing, Amiens University Hospital, Amiens, FRA; 3 Ophthalmology, Robert Debre University Hospital, Paris, FRA

**Keywords:** axial length, defocus incorporated multiple segments, highly aspheric lens, myopia, myopia control, orthokeratology

## Abstract

Purpose

Myopia is a common refractive error affecting vision. This study aims to evaluate two real-world methods for myopia control: orthokeratology (ortho-k) and defocus incorporated multiple segment (DIMS) spectacles lenses.

Methods

This retrospective observational multicenter study was conducted on a French population with myopia, treated with either DIMS spectacle lenses or ortho-k for myopia control. Baseline axial length (AL) and spherical equivalent refraction (SER) were recorded, along with data at six and 12 months. AL change was used as the primary criterion for myopia control.

Results

We included 111 patients in the ortho-k group and 88 in the spectacle lenses group. The mean baseline AL was 24.46 mm in the spectacle lenses group and 25.05 mm in the ortho-k group. No significant difference in AL elongation was observed between the two groups at the six- and 12-month time points. At six months, SER change in the DIMS group was significantly lower than in the ortho-k group; however, this difference was not maintained at 12 months. The mean AL elongation at 12 months was 0.12 mm (n=162) in the ortho-K group vs. 0.16 mm in the DIMS group (n=70).

Conclusion

The study provides new real-world data on myopia control techniques in French children. Both techniques appear to yield similar results over a one-year period.

## Introduction

Myopia has been growing significantly worldwide and is evolving into a true epidemic. Currently, approximately 30% of the global population is affected by myopia, with projections indicating that this number may rise to 50% by 2050 [1]. In France, the prevalence of myopia among children aged two to 12 years is estimated to be 15.5% [2] with an overall prevalence projected to reach 39% by 2050 [3]. Uncorrected myopia is responsible for distance vision impairment. High myopia increases the risk of various eye diseases, such as retinal detachment, myopic choroidal degeneration, myopic choroidal neovascularization, and glaucoma [4]. Given the progressive nature of myopia over time, controlling its progression is essential to reduce the risk of associated ocular complications.

Several myopia control options have been proposed, including atropine drops, defocalizing soft contact lenses, defocus incorporated multiple segments (DIMS) or Highly Aspherical Lenslet Target (HALT) spectacle lenses, and overnight orthokeratology (ortho-k) [5].

Myopia control spectacles, which employ the concept of active emmetropization, represent a promising approach. The concept utilizes a central focal point in the lens to correct myopia, while multiple peripheral hyperopic focal points help induce a signal that inhibits axial elongation of the eyeball. Lam et al. [6] demonstrated that DIMS spectacle lenses slow SER progression by 52% (n=79) and axial length (AL) elongation by 62% (n=79) in an Asian population. HALT spectacles have also proven their efficacy [7].

The ortho-k lens is another increasingly popular technique for controlling myopia progression [8]. These special contact lenses, worn at night, reshape the corneal epithelium to create a flatter central region and a more rounded peripheral region, thereby inducing a myopic defocus. Non-randomized controlled trials have reported that AL elongation in ortho-k users was slowed by 45% (n=435) compared to spectacle wearers [9]. Ortho-k lenses are associated with high compliance and provide better daytime vision quality than spectacles, as they eliminate the need for optical correction during the day.

Atropine eye drops (0.01-0.05%) can also reduce myopia progression, though the mechanism is not fully understood. Recent studies suggest that combining atropine with other techniques may enhance its efficacy [10-13].

Despite these options, there are no established guidelines for treating myopia progression. To our knowledge, there are reports of spectacles lenses and atropine use and also overnight ortho-k in real life for myopia control in the literature. However, most clinical studies report results from Asian populations, which may not reflect outcomes in European populations; major studies on HALT and DIMS spectacles may also be sponsored by the companies that manufacture the lenses.

Additionally, the choice of the treatments depends on their availability, the reimbursement process, and the age of the patient, particularly in relation to the use of contact lenses.

Our study aims to evaluate DIMS spectacle lenses and ortho-k in slowing myopia progression in a French population, assessing their effectiveness over a one-year period.

## Materials and methods

Study design

This multicenter retrospective study included patients with myopia evaluated in both university-based hospitals and private practices in the northwest region of France. Data was collected from June 2021 to June 2022. The study was approved by the institutional review board (reference PI20228430XXX) and conducted in accordance with the Declaration of Helsinki. Local ethics committee approval was obtained.

Inclusion criteria

Inclusion criteria included patients diagnosed with myopia, defined as a cycloplegic SER of -0.25 diopters or worse, aged 22 years or younger, using myopia control techniques such as ortho-k or DIMS spectacles, and having available refraction and AL data recorded at intervals of at least six months.

Exclusion criteria

Exclusion criteria included the absence of an initial AL measurement at baseline or at any follow-up point, myopia due to genetic syndromes (e.g., Marfan or Stickler syndrome), and patients with other ocular conditions (e.g., glaucoma, juvenile cataract, retinal diseases, or any form of strabismus). The treatment choice between ortho-k lenses and DIMS spectacles was based on the preferences of patients and their families.

Procedure

Age, sex, and SER errors were documented to minimize enrollment bias. If myopia was bilateral, both eyes of the patient were included in the analysis.

Each patient was followed by the same ophthalmologist and orthoptist throughout the study. Baseline comprehensive eye exams included cycloplegic refraction and visual acuity (VA) testing. Refractive error was measured using the NIDEK Tonoref III (NIDEK, Gamagori, Japan), and cycloplegia was induced with three drops of cyclopentolate hydrochloride at 10-minute intervals. Refractive error was determined by auto-refraction followed by subjective refraction. SER was calculated as spherical power plus half the cylindrical power. As ethnic data collection is prohibited in France, all participants were classified as “French” in this study including mostly Caucasians, but also Asians, and Africans.

The ortho-k lenses used were DRL® (nominal Dk of 100×10¹¹ cm²·mLO₂; Precilens, Créteil, France, https://www.precilens.com/en/orthokeratology.php). Fitting was performed by an orthoptist according to the manufacturer's guidelines. Lens parameters were adjusted based on lens centration, movement, fluorescein staining, and corneal topography (Pentacam® Oculus, Wetzlar, Germany). Participants were trained in lens application, removal, and care. After fitting, they wore the lenses for at least six hours per night. All the subjects wore their lenses the night before the day of data collection. The examinations included measurements of the subject’s VA, refraction, and AL. Follow-up exams were scheduled one day, one week, one month, and then every three to four months after the initial fitting.

In the DIMS spectacle lenses group, MiyoSmart® lenses (Hoya Corporation, Tokyo, Japan, https://www.hoyavision.com/uk/for-spectacle-wearers/miyosmart) were prescribed and fitted according to the manufacturer’s guidelines, with participants instructed to wear them as much as possible during waking hours. Follow-up exams were scheduled every six months.

The primary outcome variables were changes in SER and AL. Cycloplegic autorefraction was performed after cyclopentolate instillation, and AL measurements were conducted with the same IOL Master 700 (Carl Zeiss Meditec, Germany) in hospitals and Lenstar 900 in private settings (Haag-Streit, Zug, Switzerland). Changes in SER and AL were recorded at six and 12 months.

Statistical analysis

Continuous variables were summarized as means and SDs, while categorical data were expressed as frequencies and percentages. Student's t-tests were used to compare gender distribution between groups.

A generalized estimating equations (GEE) model was employed to evaluate the treatment effects on changes in SER and AL relative to baseline. The dependent variables were defined as changes in SER and AL (ΔSER and ΔAL) from their baseline (0M) values. The model included main effects for group (ortho-K and DIMS) and time (0M, 6M, 12M), as well as an interaction effect between group and time (group×time), with age and baseline measurements (SER and AL at 0M) as covariates to adjust for baseline differences. Missing values were handled by list-wise deletion for analytical consistency. Due to the presence of missing data and potential unbalanced observations across time points, an independent working correlation matrix was specified in the GEE analysis. This approach, in combination with a robust sandwich estimator, provides reliable inference without assuming correlated residuals between time points. Bonferroni correction was applied for multiple comparisons, setting the adjusted significance level at p<0.05.

All statistical analyses were performed using SPSS Statistics for Windows, Version 17.0 (SPSS Inc., Chicago, 2008). A p-value of <0.05 was considered statistically significant.

## Results

Population analysis

The groups were defined according to the inclusion criteria: 111 patients (222 eyes) in the ortho-K group and 88 patients (176 eyes) in the DIMS spectacles group were included. Demographic and baseline clinical characteristics are described in Table 1.

**Table 1:** Characteristics of the population Results are expressed as the mean and SD (mean±SD). T-tests were used to compare between groups, and a p-value <0.05 was considered significant. Ortho-k, orthokeratology; DIMS, defocus incorporated multiple segments

	DIMS spectacle lenses (n=176)	Ortho-k (n=222)	T-value	P
Age	11.24±2.19	13.61±- 2.87	-7,34	<0.01
Sex-ratio M/F	50%/50%	33.3%/66.7%	3,37	<0.01
Baseline myopia (D)	-2.25±-1.64	- 3.84±-2.61	-7,36	<0.01
Baseline axial length (mm)	24.46±0.95	25.05±-1.11	-5,67	<0.01
Mean follow-up (months)(range)	9.40±4.41, 6-24	17.13±7.92, 6-36	-12,28	<0.01
Progression >0.5 diopter/year before treatment	74 (42%)	102 (46%)	-5,75	0.43

Since participants were not randomly allocated to groups, significant baseline differences were observed. Pairwise comparisons showed the following statistically significant (p<0.05) differences: patients in the ortho-K group were significantly older (13.61 vs. 11.24 years, p<0.01), had a higher proportion of females (66.7% (n=222) vs. 50% (n=176), p<0.01), and presented with a higher SER (-3.84 vs. -2.25 D, p<0.01) and longer AL (25.05 vs. 24.46 mm, p<0.01) than those in the DIMS group. However, the GEE analyses adjusted for these baseline differences; Prior to the myopia control treatment, the proportion of moderate and fast progressors (progression >0.5 diopter/year) were similar between groups (p=0.43). No adverse events were observed in any group during the study.

Primary outcome: changes in spherical equivalent refraction and axial length

SER progression was -0.31 D±0.53 (n=104, p=0.02) at six months and -0.35 D±0.57 at 12 months in the ortho-K group (n=162, p<0.01), compared to -0.12 D±0.37 (n=150, p<0.01) and -0.23 D±0.38 (n=71, p<0.01) in the DIMS group at six and 12 months, respectively. In the DIMS group, 23 out of 176 patients (13%) showed no SER progression, compared to 88 out of 233 (37.7%) in the ortho-K group.

Mean AL elongation was 0.06±0.17 mm (n=104, p=0.02) at six months and 0.12±0.22 mm (n=162, p<0.01) at 12 months in the ortho-K group, compared to 0.09±0.14 mm (n=150, p<0.01) and 0.16±0.16 mm (n=70, p<0.01) in the DIMS group at six and 12 months, respectively. Among the DIMS group, 11 of 176 patients (6.5%) showed no AL elongation over one year, compared to 26 of 222 patients (11.7%) in the ortho-K group (see Table 1).

**Figure 1:**
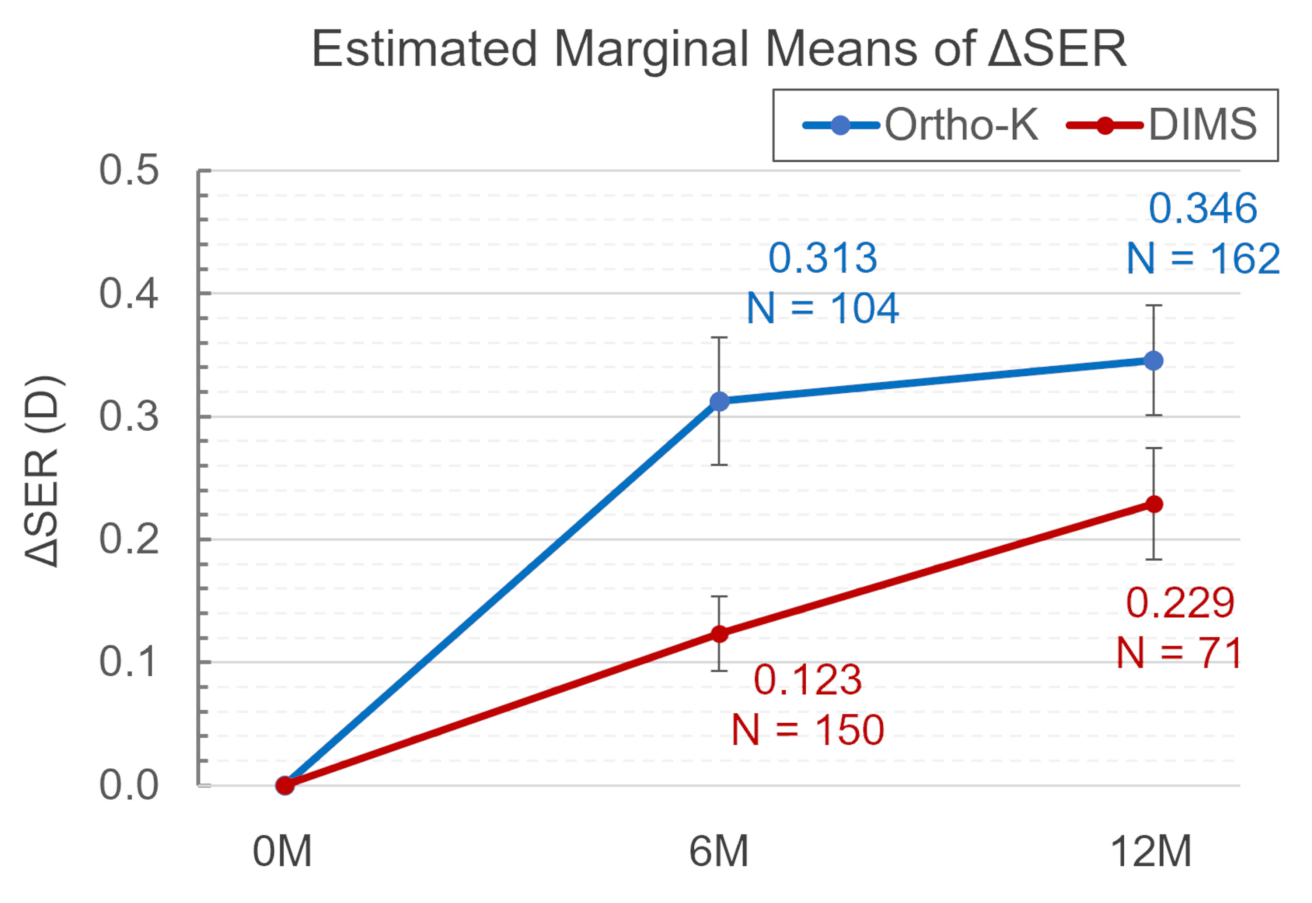
Model-adjusted mean difference SER (ΔSER) from baseline to 12 months in the ortho-K (blue) and DIMS (red) groups The error bars represent the SE, calculated as the SD divided by the square root of the sample size. The sample size (N) for each time point is indicated below the corresponding data point. SER, spherical equivalent refraction; SE, standard error; ortho-k, orthokeratology; DIMS, defocus incorporated multiple segments

According to the GEE model (see Table 2 and Figure 1), the baseline SER was adjusted to an estimated marginal mean (EMM) of 3.117, with a significant group difference in adjusted ΔSER (p=0.004). Specifically, at six months, the EMM (EMM) for ΔSER in the DIMS group was significantly lower than in the ortho-K group (0.123 vs. 0.313, p=0.024). However, the difference in adjusted ΔSER between the two groups was not significant at 12 months (0.229 vs. 0.346, p>0.05).

**Table 2:** Estimated marginal means of ΔSER (D) The data are presented as mean±SD. Treatment effects on changes in SER were evaluated using GEE. A p-value <0.05 was considered significant. Covariates included in the model are fixed at the following values: age=12.50; ref=3.117. GEE, generalized estimating equations; SER, spherical equivalent refraction; ortho-k, orthokeratology; DIMS, defocus incorporated multiple segments

Estimated marginal means of ΔSER (D) over time for each group			
(I) Group@Time	(J) Group@Time	Mean±SD (95% CI: lower;upper)	Sig.
Ortho-K@0M	Ortho-K@6M	-0.313±0.052 (-0.464;-0.161)	0.000
	Ortho-K@12M	-0.346±0.045 (-0.477;-0.214)	0.000
Ortho-K@6M	Ortho-K@12M	-0.033±0.048 (-0.175;0.108)	1.000
DIMS@0M	DIMS@6M	-0.123±0.030 (-0.213;-0.034)	0.001
	DIMS@12M	-0.229±0.045 (-0.362;-0.096)	0.000
DIMS@6M	DIMS*12M	-0.106±0.044 (-0.234;0.023)	0.239
Estimated marginal means of ΔSER (D) by group comparison at each time point			
Ortho-K@6M	DIMS@6M	0.189±0.060 (0.013;0.365)	0.024
Ortho-K@12M	DIMS@12M	0.117±0.064 (-0.070;0.304)	1.000

**Figure 2:**
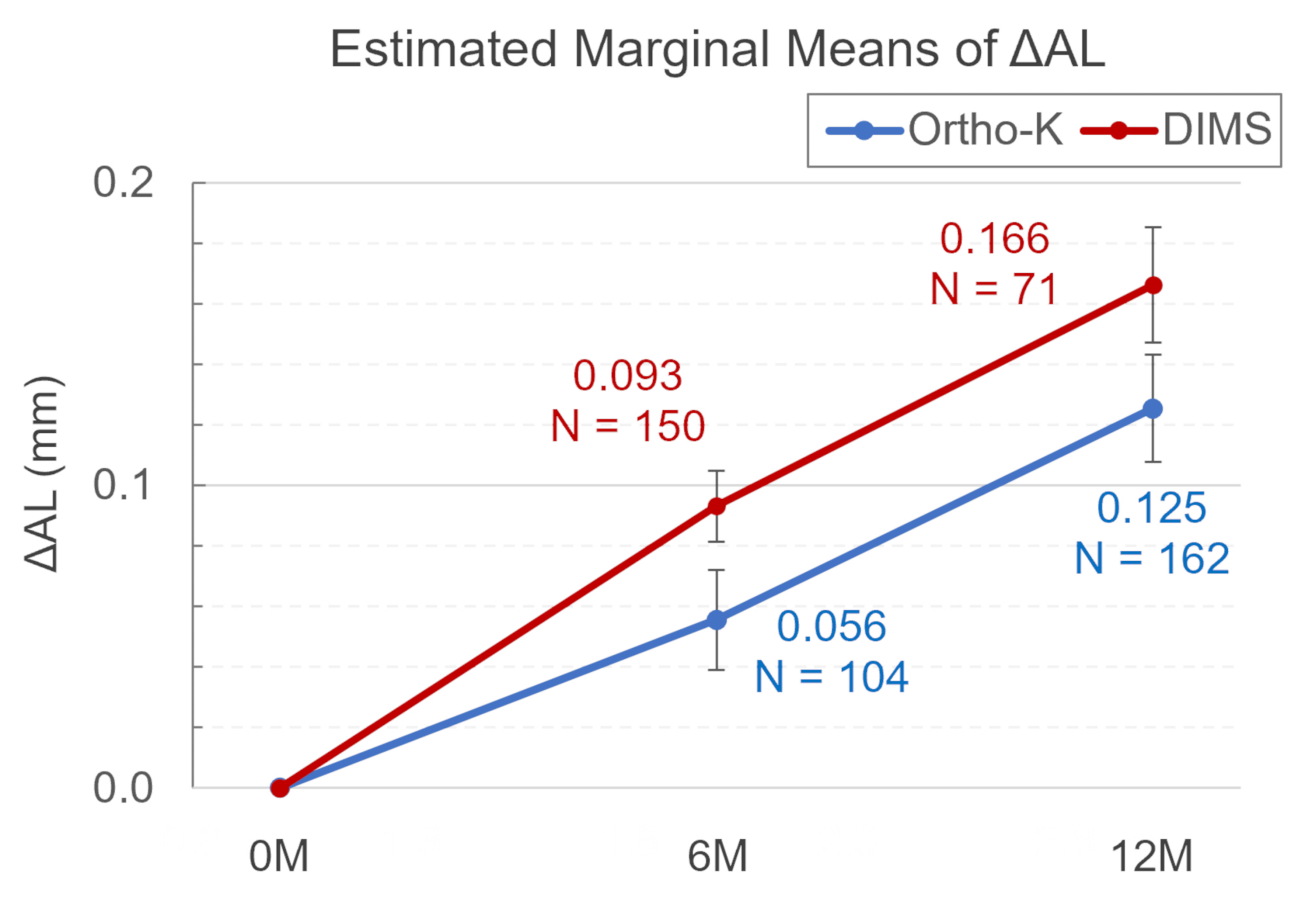
Model-adjusted mean difference AL (ΔAL) from baseline to 12 months in the ortho-K (blue) and DIMS (red) groups The error bars represent the SE, calculated as the SD divided by the square root of the sample size. The sample size (N) for each time point is indicated below the corresponding data point. Ortho-k, orthokeratology; DIMS, defocus incorporated multiple segments; SE, standard error

For AL, with a baseline fixed at 24.767, AL increased over time in both groups (see Table 3 and Figure 2). A significant main effect of the group was observed on ΔAL (p=0.031), indicating that overall the ortho-K and DIMS groups exhibited significantly different ΔAL across time points.

**Table 3:** Estimated marginal means of ΔAL (mm) The data are presented as mean±SD. Treatment effects on changes in AL were evaluated using GEE. A p-value <0.05 was considered significant. Covariates included in the model are fixed at the following values: age=12.50; ref=4.7671. GEE, generalized estimating equations; AL, axial length; ortho-k, orthokeratology; DIMS, defocus incorporated multiple segments

Estimated marginal means of ΔAL(mm) over time for each group			
(I) Group@Time	(J) Group@Time	Mean±SD (95% CI: lower;upper)	Sig.
Ortho-K@0M	Ortho-K@6M	-0.056±0.017 (-0.104;-0.007)	0.012
	Ortho-K@12M	-0.125±0.018 (-0.178;-0.073)	0.000
Ortho-K@6M	Ortho-K@12M	-0.070±0.022 (-0.135;-0.004)	0.026
DIMS@0M	DIMS@6M	-0.093±0.012 (-0.127;-0.059)	0.000
	DIMS@12M	-0.166±0.019 (-0.222;-0.111)	0.000
DIMS@6M	DIMS@12M	-0.073±0.019 (-0.128;-0.018)	0.002
Estimated marginal means of ΔAL(mm) by group comparison at each time point			
Ortho-K@6M	DIMS@6M	-0.038±0.020 (-0.097;0.022)	0.959
Ortho-K@12M	DIMS@12M	-0.041±0.026 (-0.177;0.035)	1.000

However, the non-significant group×time interaction effect (p=0.089) suggests that the difference between treatment groups does not vary significantly at individual time points (Table 4).

**Table 4:** Tests of model effects The intercept reflects the estimated mean change from baseline and does not represent between-group differences.

Source	Type III			
	Wald Chi-Square	df	Sig.	
Dependent variable: ΔSER				
Intercept	92.026	1	0.000	
Group	8.439	1	0.004	
Time	94.256	2	0.000	
Group×time	10.150	2	0.006	
Dependent variable: ΔAL				
Intercept	146.829	1	0.000	
Group	4.654	1	0.031	
time	150.579	2	0.000	
Group×time	4.847	2	0.089	

## Discussion

Our study provides new data on the effectiveness of DIMS spectacle lenses and overnight ortho-k in a European population. The findings suggest that both myopia control spectacles and ortho-k appear to be effective techniques with comparable outcomes. These treatments could, therefore, be considered primary options for myopia control.

Effectiveness of the myopia control techniques

The study results indicate that both ortho-K and DIMS lenses effectively slow myopia progression, as shown by AL measurements, and yield comparable outcomes in a European population after one year. This aligns with findings from pivotal studies on these treatments [6,8,9].

The study sample primarily consisted of “French” patients, including only Caucasians. However, it is worth noting that myopia progression rates are often higher in Asian populations, which could affect the generalizability of these results. It is essential to consider this when comparing figures in the literature, particularly in the case of Asian patients, as several studies [14,15] have demonstrated that Asian patients exhibit a higher prevalence of progressive myopia compared to Caucasians (approximately +0.2 diopters more per year).

In our study, the proportion of patients showing no AL progression was 6.5% (n=176) in the DIMS group and 11.7% (n=222) in the ortho-K group. This result is comparable to the study by Lam et al., which reported that 14% (n=79) of children wearing DIMS lenses showed no AL elongation over two years [6] though it is lower than the 28% (n=54) reported by Bao et al. in their study on aspherical lenslet spectacles [16].

Another measure of treatment effectiveness is the percentage reduction in AL progression. Lam et al. randomized, double-blind clinical trial [6] demonstrated a 52% (n=79) reduction in AL elongation in children fitted with DIMS lenses, with an AL increase of approximately 0.11 mm over one year. Our findings showed a mean AL elongation of 0.16 mm in the DIMS group at 12 months. A study by Nucci et al. [17] on a European cohort reported that the DIMS group had significantly less progression than the control group. Notably, the relatively linear progression observed in the DIMS group was consistently reflected across multiple statistical approaches, including the GEE model and the supplementary two-way ANOVA analysis. This concordance suggests that the observed pattern is a characteristic of the data rather than an artifact of smoothing or imputation.

For ortho-k, our results align with major studies such as the ROMIO [8] and LORIC [18] studies. In these studies, children wearing ortho-K lenses showed AL elongation of 0.16 mm and 0.20 mm over one year, respectively. A real-world study by Holmes et al. [19] found a similar AL increase of 0.18 mm in children using ortho-k lenses. In a recent French study, the DRL ortho-K lens demonstrated an AL increase of 0.06 mm at six months and 0.12 mm at 12 months, comparable to our findings [20].

Although a statistically significant group effect on ΔAL was observed in this study (Table 4), the magnitude of the difference was only 0.04 mm, within the test-retest variability of optical biometers (typically 0.02-0.05 mm). In addition, both the GEE model and the two-way ANOVA showed no significant group×time interaction, suggesting that the between-group differences at individual time points were not statistically significant. Therefore, this result should be interpreted with caution, as such a small difference may not reflect true physiological progression and warrants further investigation in a larger sample.

Adverse events

In our study, there were no adverse events requiring discontinuation of DIMS or ortho-k. Indeed, patients with ortho-k can suffer from corneal ulceration or bacterial infection [21]. Furthermore, both techniques can lead to a deterioration in optical quality, with a loss of contrast, glare, or ghosting [22]. Moreover, there is a rebound effect of myopia treatments when discontinued, as previously shown in a meta-analysis [23]. It seems to be less common in patients treated with optical methods than with pharmacological methods.

Limitations of our study

The study’s retrospective design and potential biases (selection bias or confounding factors) should be noted when interpreting the results. This study does not include a control group, limiting comparisons to baseline data. Additionally, only DIMS lenses were evaluated, as they were more readily available than HALT lenses in our patient population. Although both DIMS and HALT use myopic defocus principles, a recent study by Guo et al. [24] suggests a slight difference in effectiveness between the two techniques.

Additionally, we did not include patients with ATROPINE, which was not available in our hospital. Only a few patients had this treatment, which was delivered from another hospital. 

Furthermore, the patient groups were not fully comparable. The ortho-K group was older, had higher baseline myopia, had longer follow-ups, and included more females. The old age of this group will likely mean that the progression will be less. This may be due to the challenges younger children face in adapting to contact lenses. The complexity of ortho-k follow-up care may explain why these patients were more likely to be seen in hospitals rather than private practices, where DIMS lenses were predominantly used. The “Procedure” section provides detailed information on the fitting protocol for ortho-k lenses. However, a compliance questionnaire was not performed, which could be another limitation.

Due to the retrospective nature of this real-life study, the timing of follow-up appointments was not scheduled, which may also be a potential measurement bias. Indeed, there is a diurnal variation in AL as shown by Chakraborty et al. [25]. Moreover, AL measurements were performed with different devices between the groups, although the same device was used during the follow-up. We also acknowledge the lack of detailed statistical analysis and the short follow-up period, which limit the robustness and generalizability of the conclusions.

Although we adjusted for baseline age and refractive error using a GEE model, the use of propensity score matching (PSM) could be considered in future studies to further reduce group imbalance, particularly if larger datasets are available. 

## Conclusions

Currently, several techniques are available to slow myopia progression, based on the principles of myopic defocus and active emmetropization. For children with progressive myopia, prompt treatment can help reduce the risk of associated ocular complications.

Both DIMS spectacle lenses and ortho-k demonstrated comparable efficacy in controlling myopia progression over one year in a French pediatric population. Observed differences in AL elongation fell within expected measurement variability and are unlikely to be clinically meaningful.
